# Design of a novel compact highly selective wideband bandstop RF filter using dual path lossy resonator for next generation applications

**DOI:** 10.1371/journal.pone.0273514

**Published:** 2022-10-31

**Authors:** Muhammad Abdul Rehman, Sohail Khalid, Bilal Mushtaq, Muhammad Idrees

**Affiliations:** Electrical Engineering Department, Riphah International University Islamabad, Islamabad, Pakistan; Edinburgh Napier University, UNITED KINGDOM

## Abstract

This article presents the design of a wideband bandstop RF filter intending to improve selectivity and compactness. Conventional bandstop filter topology with finite unloaded quality factor produces degraded bandstop filter performance due to dissipation loss. In the proposed filter design, a novel dual path Capacitive Coupled Step Impedance Resonator (CCSIR) structure is used to obtain an infinite stopband attenuation. A uniform impedance resonator is used in Path 1, whereas Path 2 contains a resonator that is twice coupled to the transmission lines. The electrical length of both paths is chosen to be out of phase, resulting in a high rejection level at higher frequencies. It has been analyzed that the selectivity can be improved by increasing the order of the dual coupled step impedance resonator. The proposed design produces a wideband BSF centered at 5.25 GHz with a high rejection level of 104.3 dB and fractional bandwidth of 58.5%. The results have demonstrated that the resonant frequencies are regulated by varying the electrical length of CCSIR. Moreover, it has been realized that out of phase signal cancellation due to the dual path is involved in producing the finite frequency transmission poles, which further enhances the filter selectivity. However, the same electrical performance can only be achieved from coaxial cavities and waveguides due to the high-quality factor. The proposed topology is fabricated and measured on a high-frequency microstrip substrate having a low-quality factor with a compact output and better electrical performance compared to coaxial cavities or waveguides. Due to its high electrical performance and small size, the proposed BSF is appropriate for 4G and 5G (FR1) applications. The measurement shows good concurrence with the full-wave EM simulated results. The fabricated prototype of third order BSF has a compact size of (0.7 × 0.77)λ_*g*_ at 5.25 GHz.

## 1 Introduction

Wireless communication systems may encounter a plethora of impurities. Non-linearities in hardware components or several carriers within the same data links are the main sources of impurities. These impurities are referred to as passive intermodulation. Many solutions have been formulated to resolve these disputes [1–24]. One approach uses signal interference and cross-coupling to design a wideband Band Stop Filter (BSF) [1]. In another one, a wideband BSF is designed using two $\frac{\lambda }{4}$ open and circuited transmission couple lines [2]. The proposed filter shows optimal electrical performance. However, circuit dimensions are quite compromised. To design a miniaturized BSF, parallel-coupled lines and loaded stubs are used in [3]. The designed filter shows good out-of-band insertion with four transmission poles. Though, only one transmission zero indicates the week rejection of the signals. Furthermore, a wideband BSF is developed using dual-coupled resonators [4]. However, the additional capacitors may cause an undesired parasitic effect at high frequencies. In [5], dual-section open stubs are used to design a highly selective BSF. However, the circuit size remains an issue. Additionally, a complementary split-ring resonator is used to design a wideband bandpass filter [6]. The proposed filter is compact. However, the electrical performance is compromised. Moreover, Band-stop filters have been designed by employing symmetrical three-layer ring structures, each with a high-impedance phase shift section, connected lines, and short-stubs [7]. The proposed filter has a relatively small circuit size, but it is challenging to fabricate. In [8], a wideband BSF is proposed using two coupled line sections with loaded stubs and an inner couple line section. The proposed filter has a very good out-of-band insertion. Whereas the filter produces only one transmission zero. Another method for designing wideband BSF is to use a step impedance couple line shunt connected with an open-circuited stub [9]. The proposed filter performs well in terms of in-band rejection, while out-of-band insertion is not good. Moreover, a wideband bandstop filter is proposed using stub loaded cross-coupling technique [10]. The proposed design shows good in-band isolation; however, out of band insertion is low. Two wideband bandstop filters with multiple transmission zeros are designed in [11, 12], respectively. Furthermore, [13] describes the design of a triple mode microstrip bandstop filter using open-circuited stubs. In [14] a new miniature band-stop filter based on metamaterial resonator unit cell is presented. The circuit size is compact however the design lack good selectivity. A microstrip bandstop filter (MSBSF) intended to prevent electromagnetic information leakage in high power transmission line is presented in [15]. The electrical performance are good however circuit size is large. Due to microstrip substrate limitations, all the above reported bandstop filters cannot attain high rejection levels [16–23]. One solution is to fabricate the proposed topology using a coaxial cavity or waveguide. However, the fabrication cost and the size will be increased significantly.

**Fig 1:**
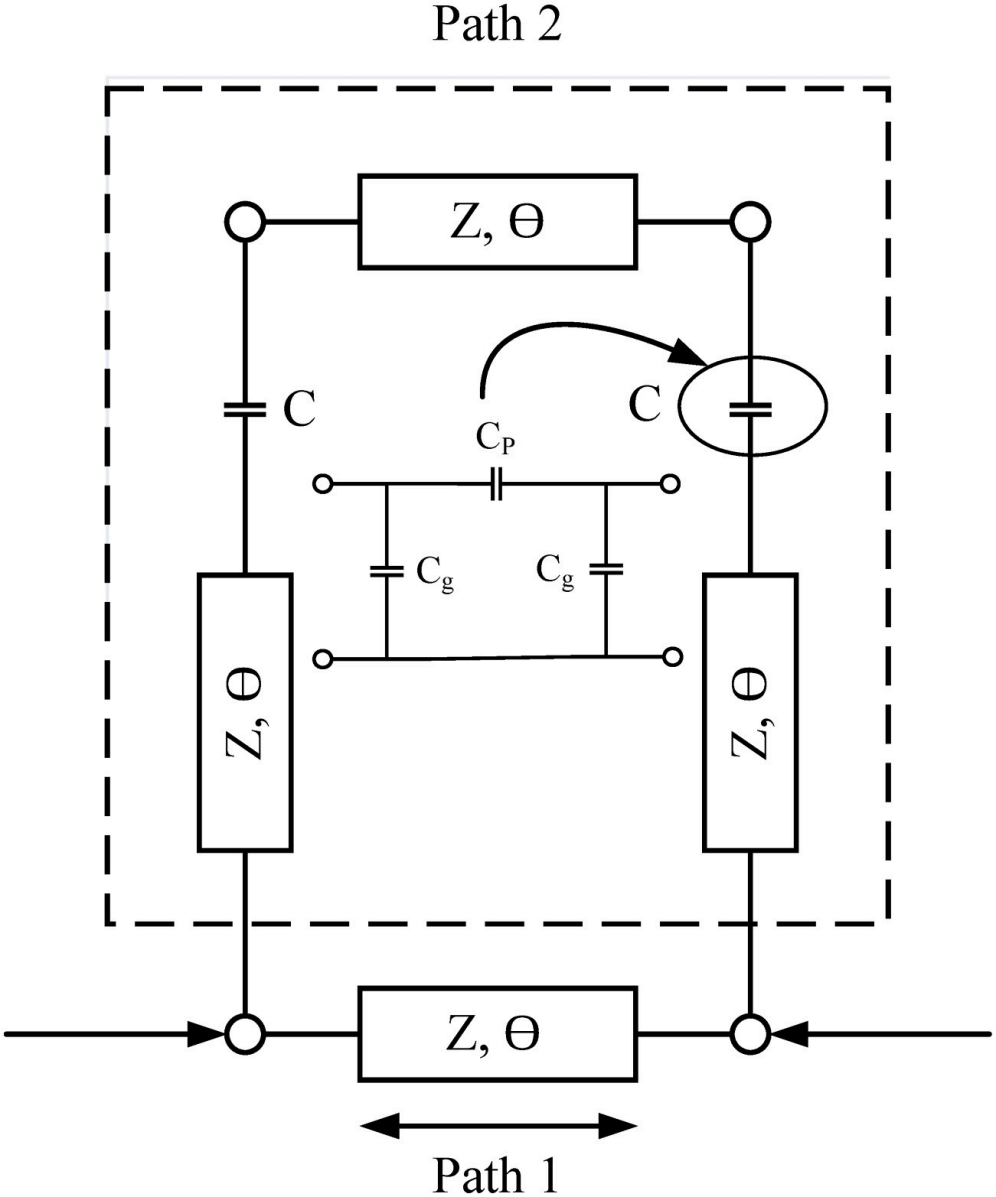
Schematic model of proposed BSF.

This paper presents a compact, highly selective bandstop RF filter using a dual path CCSIR structure as shown in Fig 1. The proposed filter produces a highly selective frequency response with a rejection level greater than 100*dB*. Analyses show that the selectivity is further improved by increasing the order of the dual coupled step impedance resonator. Moreover, the stopband is independently controlled by varying the electrical length of coupled SIR. In this configuration, the designed filter attains good electrical performance, controllable stopband frequency, compact size, and ease of fabrication.

**Fig 2:**
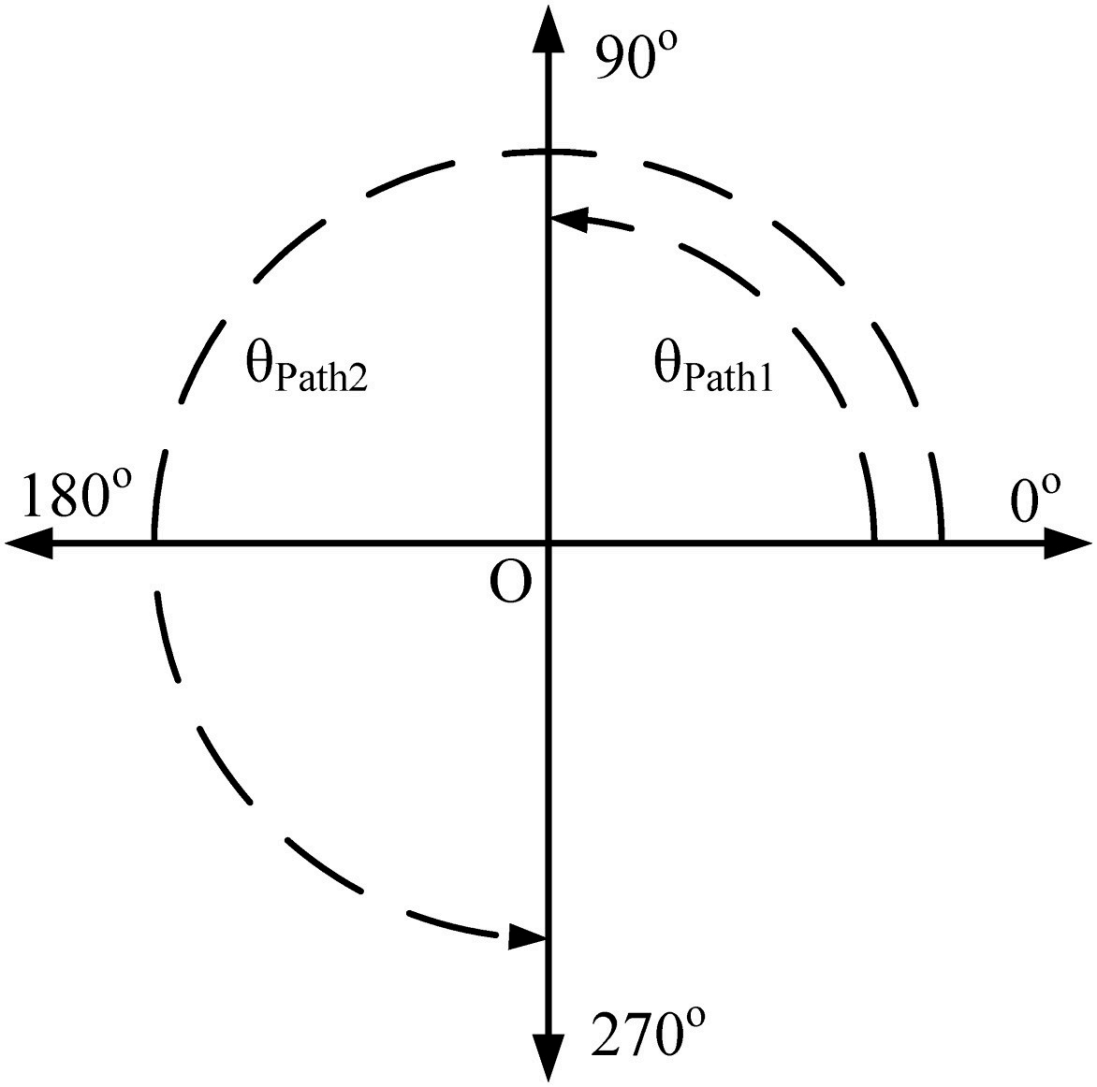
Coordinate system representantion for proposed BSF.

Fig 1 shows the schematic model of the proposed bandstop filter with two transmission paths. Path-1 entails a uniform impedance resonator with characteristics impedance “Z” and electrical length “*θ*”. However, path-2 includes a resonator, which is presented as a 1pF capacitor coupled twice with the transmission lines of impedance “Z” and electrical length “*θ*_1_” whereas *θ* = *θ*_1_. Path-1 has an electrical length of 90°, while path-2 has an electrical length of 270°. The electrical length of both pathways is deliberately chosen to be out of phase, resulting in a high rejection level at higher frequencies, as illustrated in Fig 2.

An ideal transmission line model is depicted in Fig 1. Based on this model, the filtering function is retrieved using the conversion between ABCD and Y-Parameters and vice versa. The ABCD parameters of path-2 are calculated as [25].

$$\begin{array}{c} {}[\begin{array}{c} A\;\;\;\;\;\;B\\ C\;\;\;\;\;\;D \end{array}]Path2=T_{TL}\times T_{CP}\times T_{TL}\times T_{CP}\times T_{TL} \end{array}$$ (1)

$$\begin{array}{c} T_{TL}=[\begin{array}{c} \text{Cos}(\theta )\;\;\;\;\;\;\;\;jZ\;\text{sin}(\theta )\\ \frac{j\text{Sin}(\theta )}{Z}\;\;\;\;\;\;\;\;\text{Cos}\left(\theta \right) \end{array}] \end{array}$$ (2)

$$\begin{array}{c} T_{CP}=[\begin{array}{c} 1\;\;\;\;\;\;\;C\\ 0\;\;\;\;\;\;\;1 \end{array}] \end{array}$$ (3)

After addressing Eq.1 and converting them to Y-Parameters, the overall Y-Parameters are found by adding the Y-Parameters of path-1 and path-2, respectively.

$$\begin{array}{c} Y_{Filter}=[\begin{array}{c} Y_{11}\;\;\;\;\;\;\;Y_{12}\\ Y_{21}\;\;\;\;\;\;\;Y_{22} \end{array}] \end{array}$$ (4)

Y11={13(−jsin4(θ)z+(−4z2−1)cos(θ)sin3(θ)+5jsin2(θ)z(−4z2+1)sin(θ)−2jcos4(θ)z)}×{sin(θ)z(−13jsin4(θ)z2−23sin2(θ)cos(θ)z+jcos2(θ)z2+13sin(θ)+23sin3(θ)z}−1 (5)

$$\begin{array}{c} \begin{array}{c} Y_{12}=\left\{\left(I{\text{cos}}^{2}\left(\theta \right)z+\text{sin}\left(\theta \right)\left(-z^{2}-\frac{1}{4}\right)\text{cos}\left(\theta \right)-\frac{1}{2}Iz\right)\text{cos}\left(\theta \right)\right\}\times \{(z{\text{cos}}^{3}\left(\theta \right)\\ \;\;\;\;\;\;\;\;\;\;\;\;\;+I\text{sin}\left(\theta \right)\left(z^{2}+\frac{1}{4}\right){\text{cos}}^{2}\left(\theta \right)-\frac{1}{2}z\text{cos}\left(\theta \right)-\frac{1}{4}Iz^{2}\text{sin}\left(\theta \right){)}^{-1}\} \end{array} \end{array}$$ (6)

$$\begin{array}{c} \begin{array}{c} Y_{21}=\left\{\left(I{\text{cos}}^{2}\left(\theta \right)z+\text{sin}\left(\theta \right)\left(-z^{2}-\frac{1}{4}\right)\text{cos}\left(\theta \right)-\frac{1}{2}Iz\right)\text{cos}\left(\theta \right)\right\}\times \{\text{sin}\left(\theta \right)(z{\text{cos}}^{3}\left(\theta \right)\\ \;\;\;\;\;\;\;\;\;\;\;\;\;+I\text{sin}\left(\theta \right)\left(z^{2}+\frac{1}{4}\right){\text{cos}}^{2}\left(\theta \right)-\frac{1}{2}z\text{cos}\left(\theta \right)-\frac{1}{4}Iz^{2}\text{sin}\left(\theta \right){)}^{-1}\} \end{array} \end{array}$$ (7)

$$\begin{array}{c} \begin{array}{c} Y_{22}=\{\frac{1}{4}(-8I{\text{cos}}^{4}\left(\theta \right)z+\left(8z^{2}+2\right)\text{sin}\left(\theta \right){\text{cos}}^{3}\left(\theta \right)+7I{\text{cos}}^{2}\left(\theta \right)z\\ \;\;\;\;\;\;\;\;\;\;\;\;\;+\left(-4z^{2}-1\right)\text{sin}\left(\theta \right)\text{cos}\left(\theta \right)-Iz)\}\times \{\text{sin}\left(\theta \right)(z\text{cos}{\left(\theta \right)}^{3}\\ \;\;\;\;\;\;\;\;\;\;\;\;\;\;+I\text{sin}\left(\theta \right)\left(z^{2}+\frac{1}{4}\right){\text{cos}}^{2}\left(\theta \right)-\frac{1}{2}z\text{cos}\left(\theta \right)-\frac{1}{4}Iz^{2}\text{sin}\left(\theta \right){)}^{-1}\} \end{array} \end{array}$$ (8)

From the Y-Parameters, the S-Parameters of the filter unit are calculated as.

$$\begin{array}{c} \begin{array}{c} S_{21}=\frac{-2Y_{21}Y_{o}}{(Y_{11}+Y_{o})(Y_{22}+Y_{o})-{Y_{12}}^{2}} \end{array} \end{array}$$ (9)

**Fig 3:**
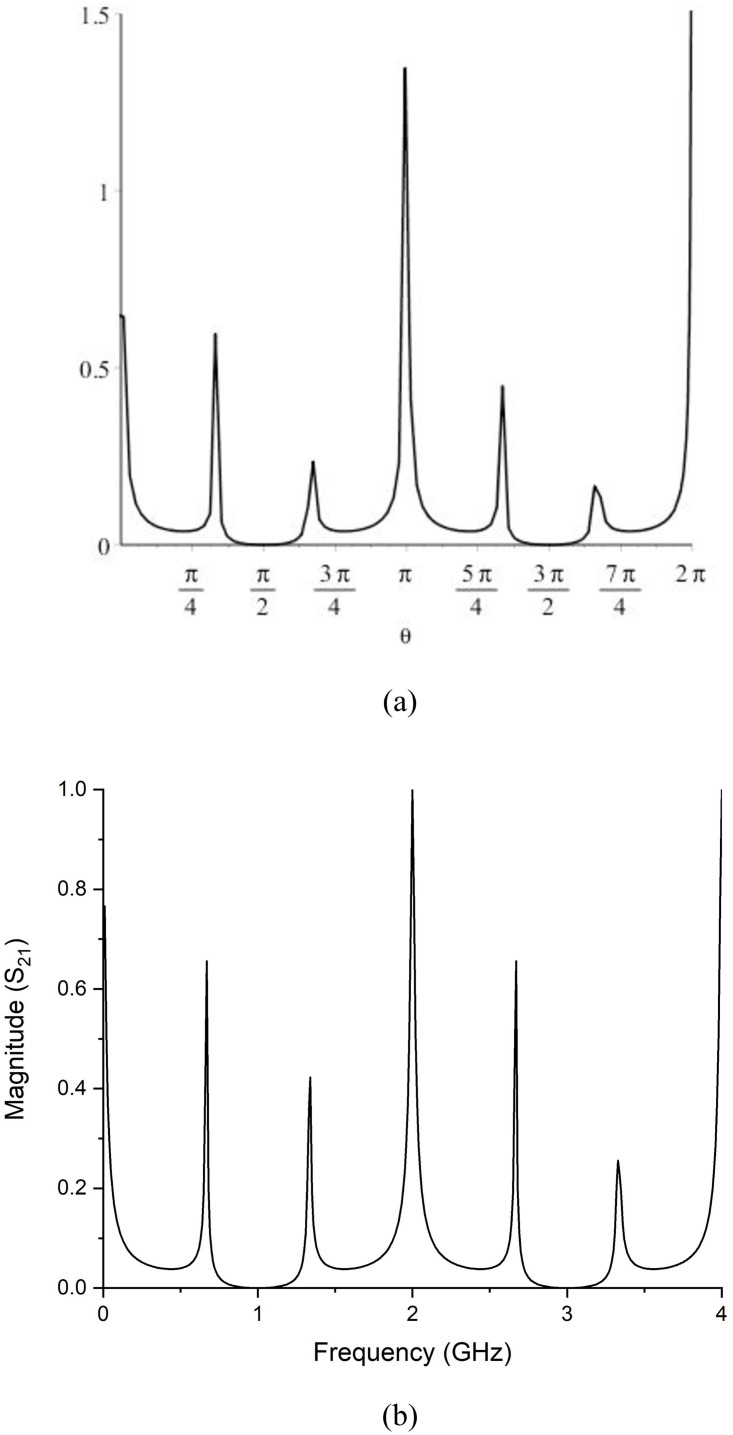
Comparison of *S*_21_ Magnitude (a) Mathematical (b) Simulated.

The magnitude of *S*_21_ is compared to the simulated results to validate the normalized transfer matrix, as shown in Fig 3. Next, the filtering function will be retrieved to examine the transfer function. For the Chevishev type frequency response, the form of the transfer function is given by [26].

$$\begin{array}{c} \begin{array}{c} {\left|S_{21}\left(\theta \right)\right|}^{2}=\frac{1}{1+\varepsilon^{2}F^{2}\left(\theta \right)} \end{array} \end{array}$$ (10)

Here F(*θ*) is the filtering function, and *ϵ* is the ripple factor, which controls the ripple-level in the passband. Eq.10 is employed to obtain the filtering function after solving Eq.9. For normalization, the capacitance is set to be 1F, and ripple level *ϵ* is also chosen to be 1.

$$\begin{array}{c} \begin{array}{c} F^{2}\left(\theta \right)=\left\{\frac{1}{4}\alpha {\text{cos}}^{8}\left(\theta \right)\beta {\text{cos}}^{6}\left(\theta \right)\gamma {\text{cos}}^{4}\left(\theta \right)\varphi {\text{cos}}^{2}\left(\theta \right)-z^{8}+2z^{4}-1\right\}\times \\ \left\{{\left(z^{2}{\text{cos}}^{2}\left(\theta \right)\left(4{\text{cos}}^{2}\left(\theta \right)z^{2}-{\text{cos}}^{2}\left(\theta \right)+1\right)\left(4{\text{cos}}^{2}\left(\theta \right)z^{2}-{\text{cos}}^{2}\left(\theta \right)-4z^{2}\right)\right)}^{-1}\right\}\\ \alpha =-16z^{8}+136z^{6}+321z^{4}+136z^{2}-16\\ \beta =40z^{8}-366z^{6}+658z^{4}-246z^{2}+40\\ \gamma =-33z^{8}+264z^{6}-351z^{4}+144z^{2}-33\\ \varphi =10z^{8}-34z^{6}+28z^{4}-34z^{2}+10 \end{array} \end{array}$$ (11)

Eq.11 shows a filtering function of the proposed topology shown in Fig 1. The driven filtering function shows that four TP’s are obtained across the stopband. The filtering function has been mapped to Chebyshev type 1 polynomials to extract electrical parameters. It is worth mentioning that the frequency-dependent terms, both in the nominator and denominator, have to be compensated with the help of electrical parameters. Therefore, the procedure presented in [24] is opted to nullify the effect of the frequency-dependent term.

**Fig 4:**
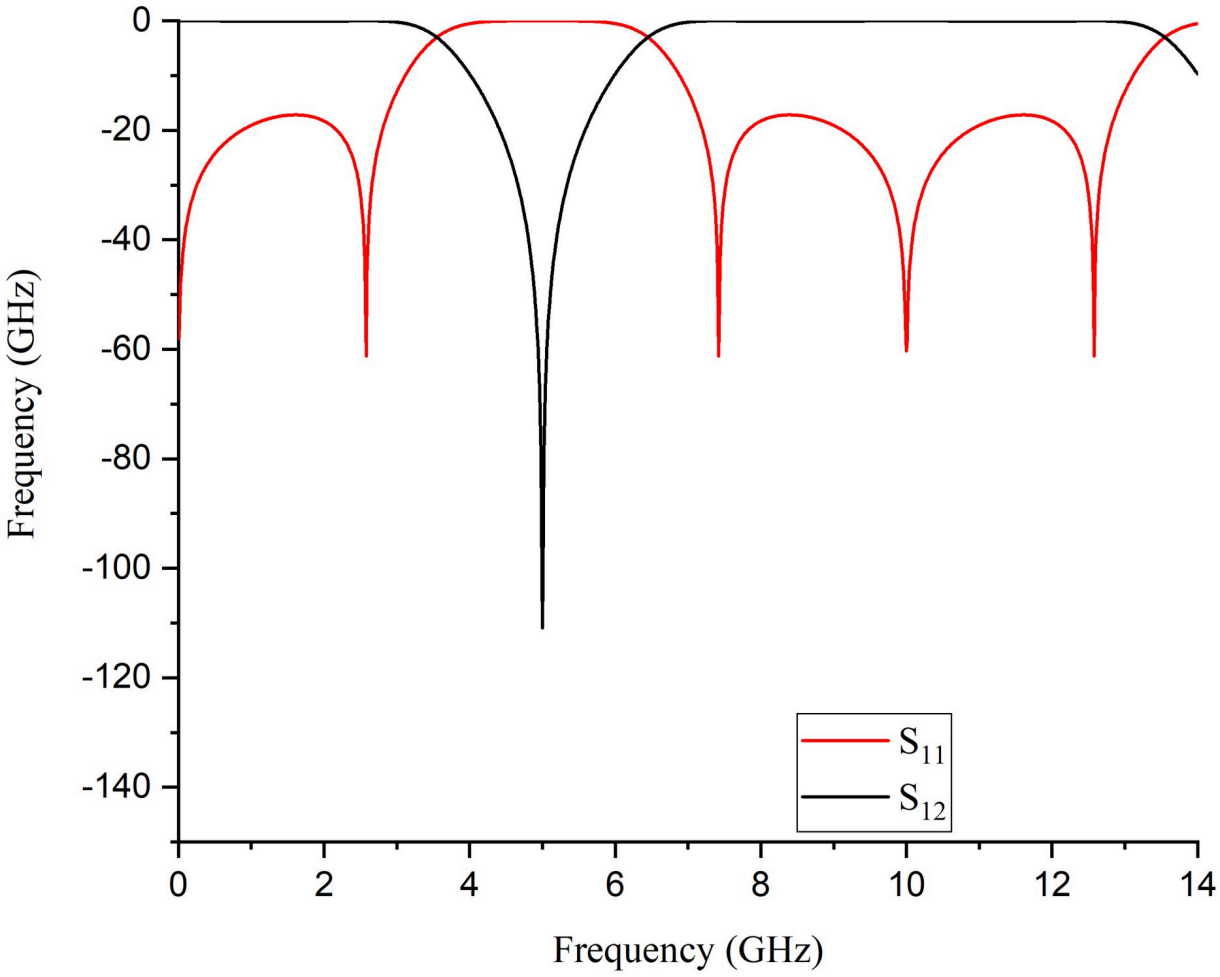
Simulated results of the ideal transmission line model.

**Fig 5:**
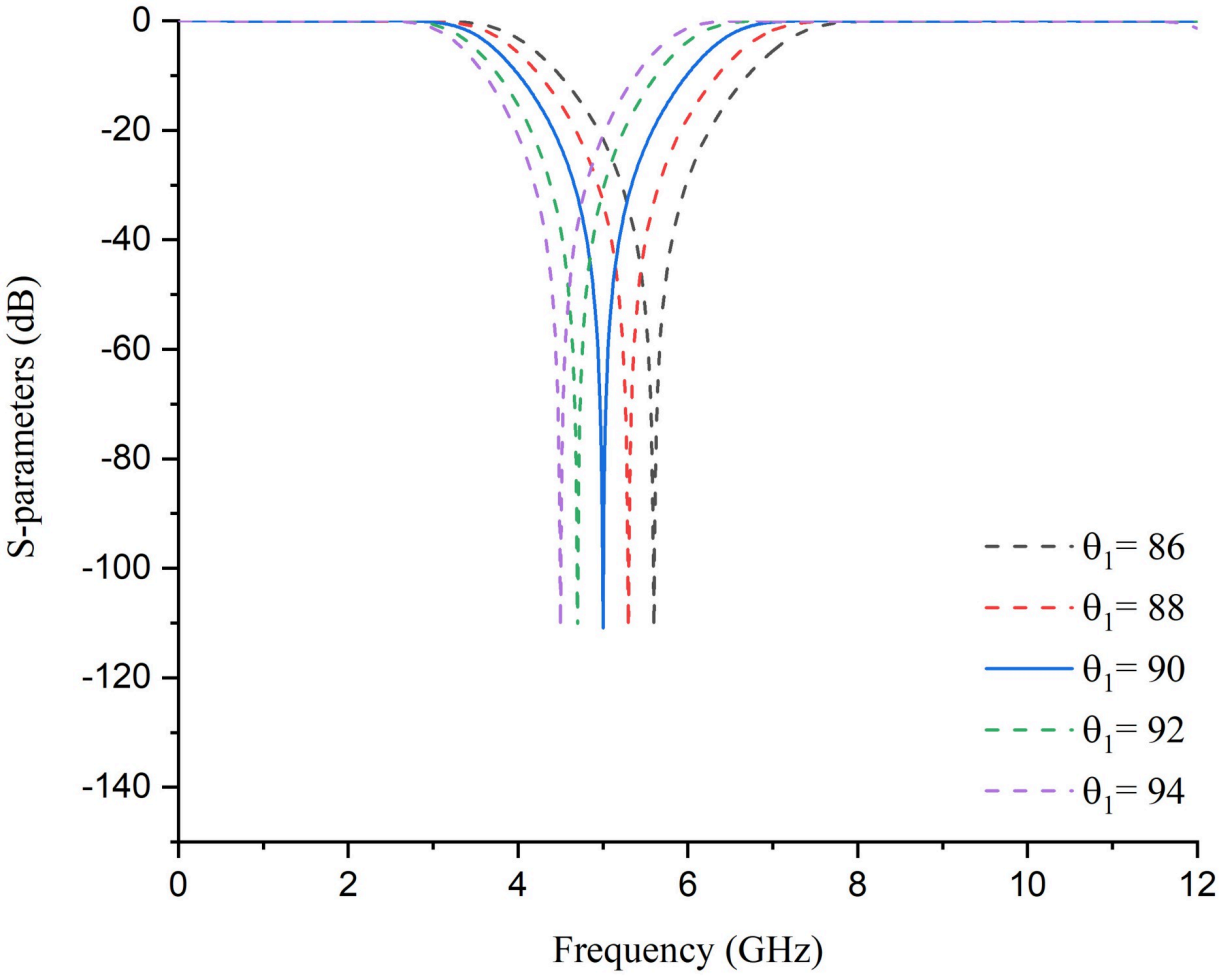
Simulated frequency response with different values of electrical length.

Fig 4 shows the S-parameter response of the ideal design topology. The detailed analysis has demonstrated that the position of transmission zero is controlled independently by changing the electrical length of path-2. As shown in Fig 5, for *θ*_1_ = 90^*o*^, the position of transmission zero is at 5 GHz. With the increase in *θ*_1_ value, the transmission zero moves towards a lower frequency. At the same time, the transmission zero moves towards the higher frequency by decreasing the value of *θ*_1_.

**Fig 6:**
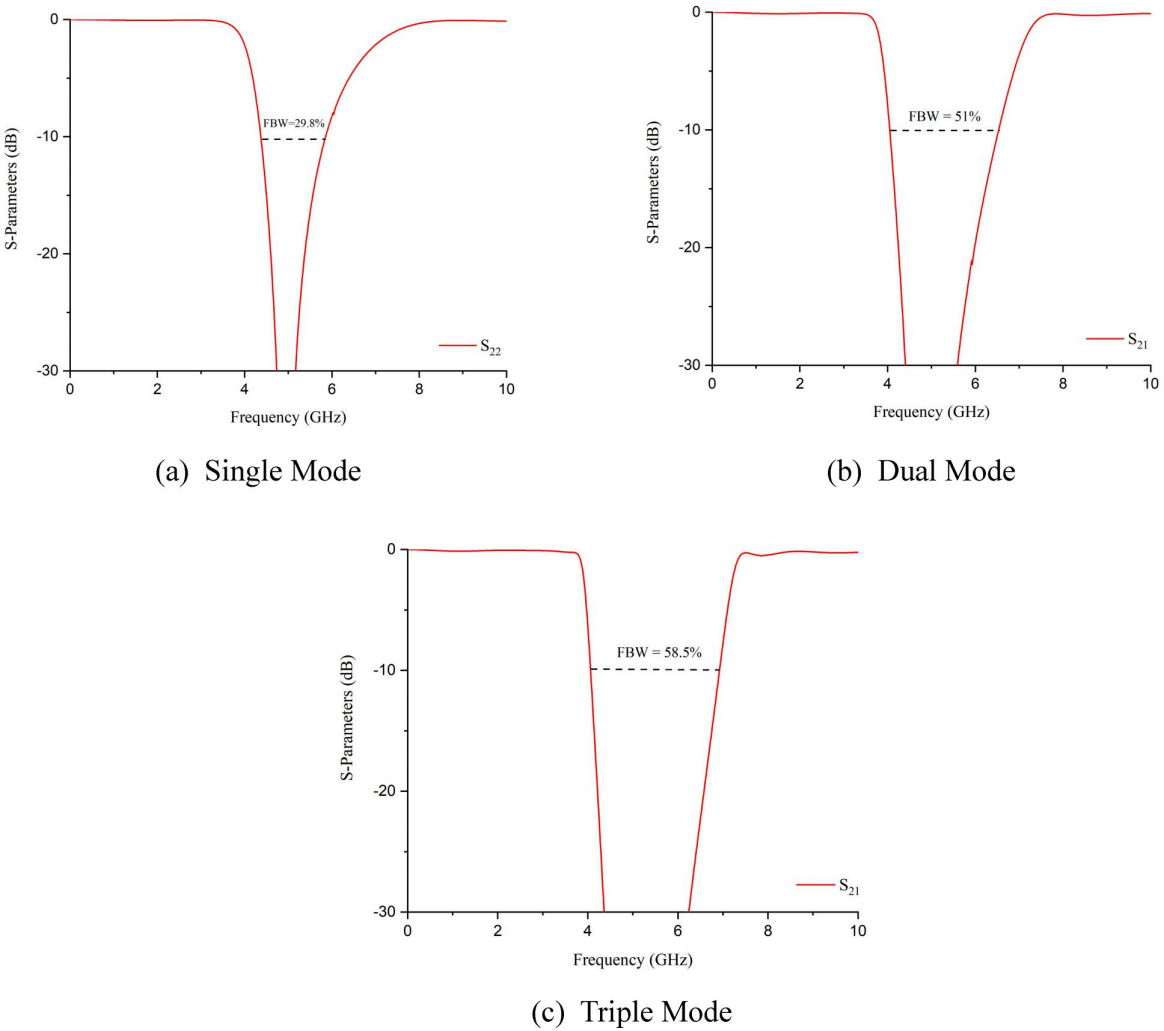
10 dB Fractional bandwidth with different transmission modes.

In addition to that, it has been seen that the modality to response includes increasing the order of the dual-path structure. By keeping the electrical parameters constant and simply altering the filter’s order, the response’s selectivity is increased significantly. It is worth mentioning that the 10 dB fractional bandwidth will also be increased with the increment in order. Fig 6 shows the response of up to a third-order structure.

**Fig 7:**
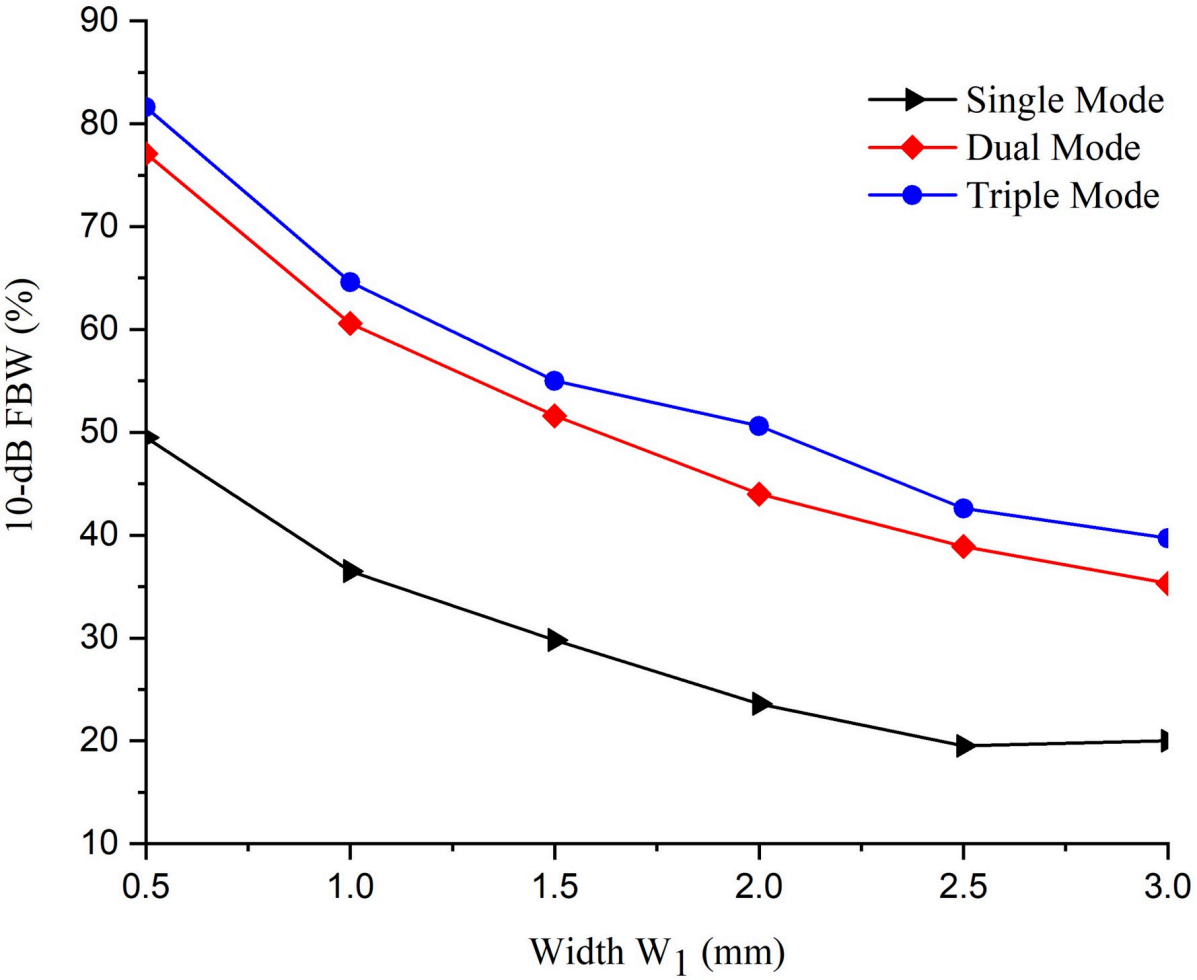
10 dB Fractional bandwidth response with variable width *W*_1_.

The response in (a) depicts the first-order filter, whereas in (b) and (c), it shows the response of the second-order and third-order filter structures. The fractional bandwidth (FBW) of the first-order filter is 29.8%, whereas the second and third-order filters contain 51% and 58.5%, respectively. Moreover, it is inferred that fractional bandwidth is independently controlled by varying the width of path-1 (*W*_1_). Fig 7 shows that *W*_1_ and FBW are inversely proportional to each other as increasing the value of *W*_1_ will decrease the FBW and vice versa.

## 2 Results

**Fig 8:**
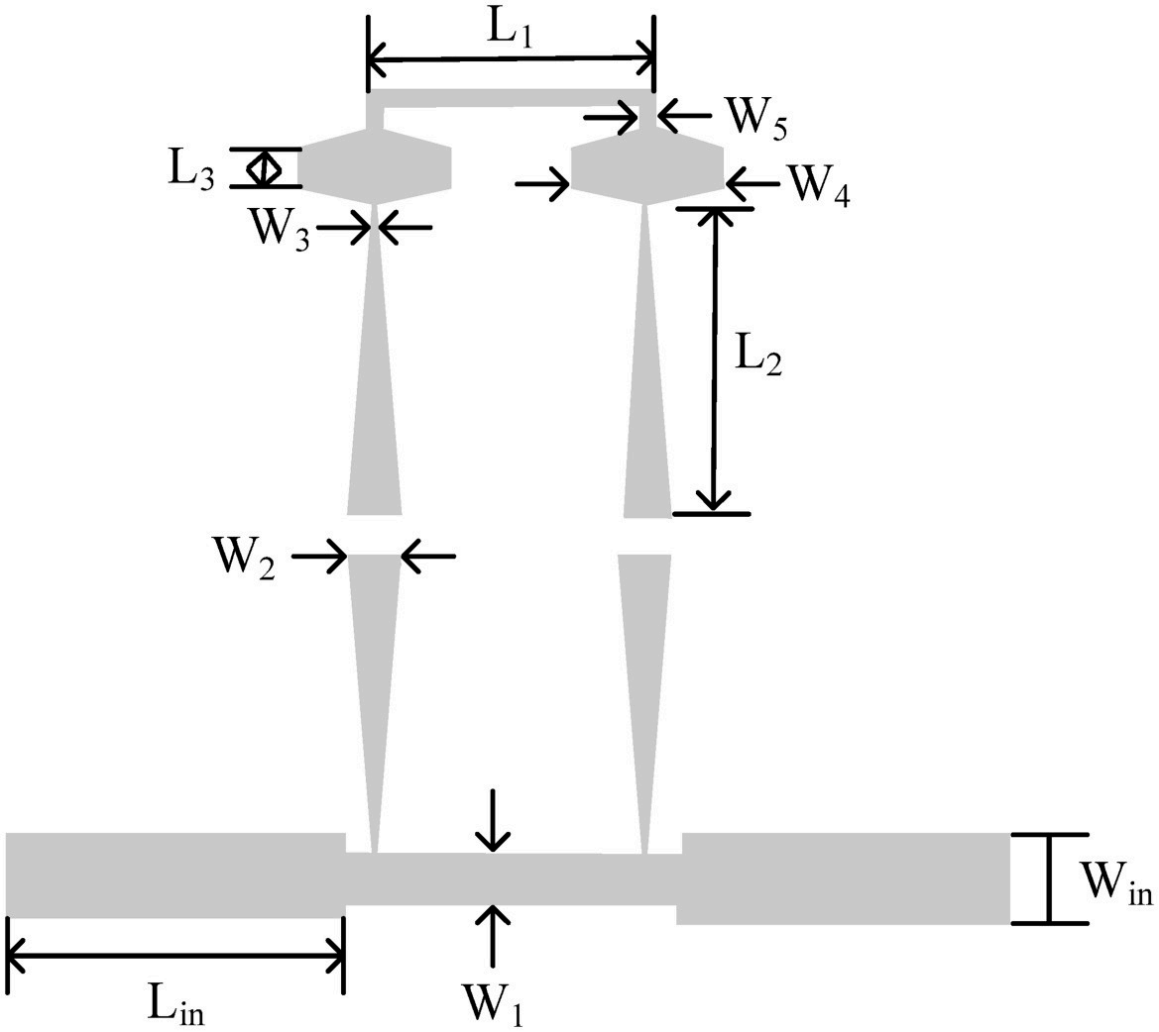
Transmission line model of proposed BSF.

For the validation of the proposed topology, three prototypes have fabricated on Roger duroid 5880 substrate having *εr* = 2.20, *δ* = 0.0009, h = 0.787, T = 0.0175mm respectively. Using the line calculator, ideal electrical parameters are converted to physical parameters,as illustrated in Fig 8, which are *W*_*in*_ = 2.6, *W*_1_ = 1.5, *W*_2_ = 1.46, *W*_3_ = 0.2, *W*_4_ = 2.96, *L*_*in*_ = 10.85, *L*_1_ = 8.68, *L*_2_ = 9.53, *L*_3_ = 1.21, *L*_4_ = 0.6, *L*_5_ = 0.9, and *G* = 1.3 (Unit in mm). Microstrip tapered lines are used to avoid the losses accrued due to the steps in transmission lines.

**Fig 9:**
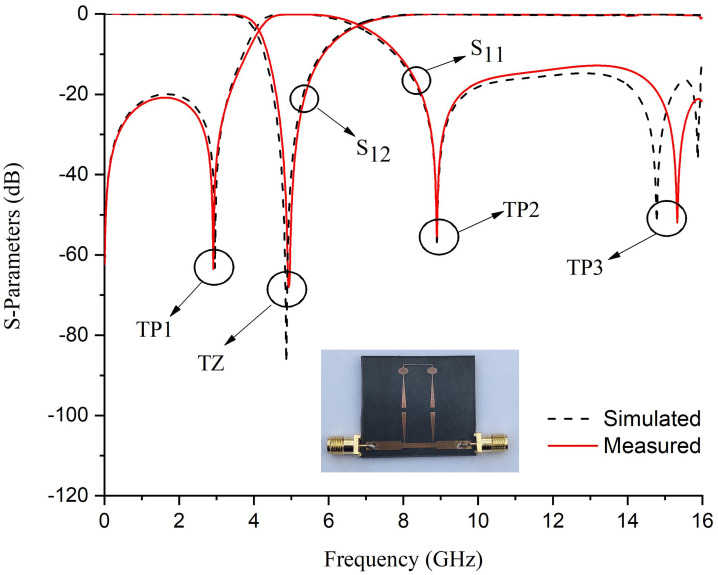
S-Parameter response of the fabricated single-order BSF.

**Fig 10:**
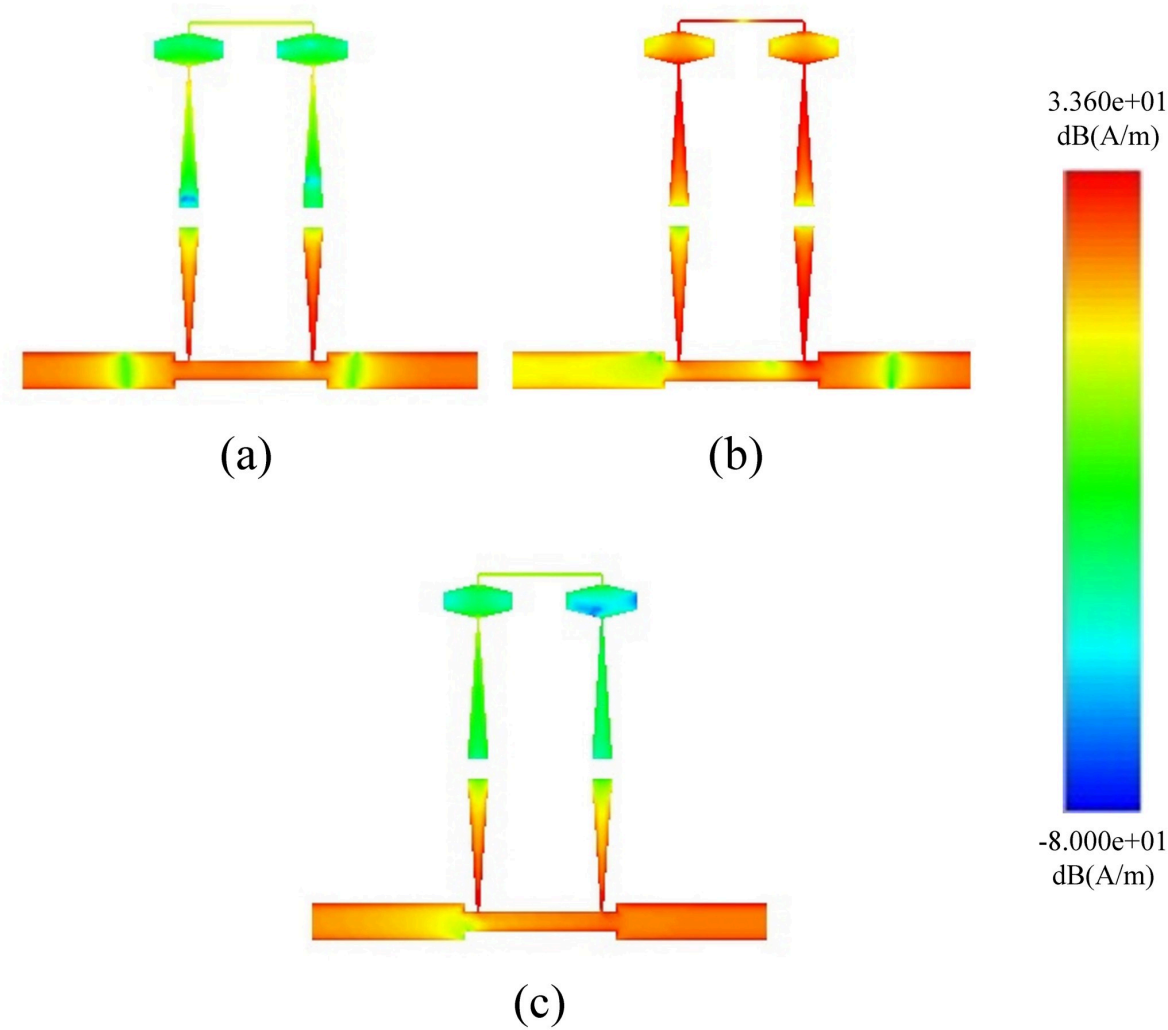
Current distribution of single order BSF.

In full-wave EM simulations, the effects of substrate loss are corrected by incorporating tuning and optimization. Theoretical analysis reveals that the destructive interference is solely responsible for the generation of a high rejection notch between the signals from both paths. Prototype 1 with its S-Parameter response is shown in Fig 9. The proposed BSF is designed and fabricated at 5.25 GHz. Four transmission poles around the stopband are achieved with 5.6 GHz to 16 GHz out-of-band insertion. The mismatch between measured and simulated results is due to substrate losses at high frequencies. Fig 10 shows the electrical field spectrum at various frequencies for the proposed BSF. Fig 10(a) and 10(c) show the electric field spectrum at passband frequencies while Fig 10(b) indictes the electric field spectrum at center frequency of the stopband. The mesh size is 80 cells/wavelength and simulated with the sweep frequency of 0.01 GHz. It depicts that at stopband frequency, both the paths contribute as a maximum field. However, at passband frequencies, the only path-1 is responsible.

**Fig 11:**
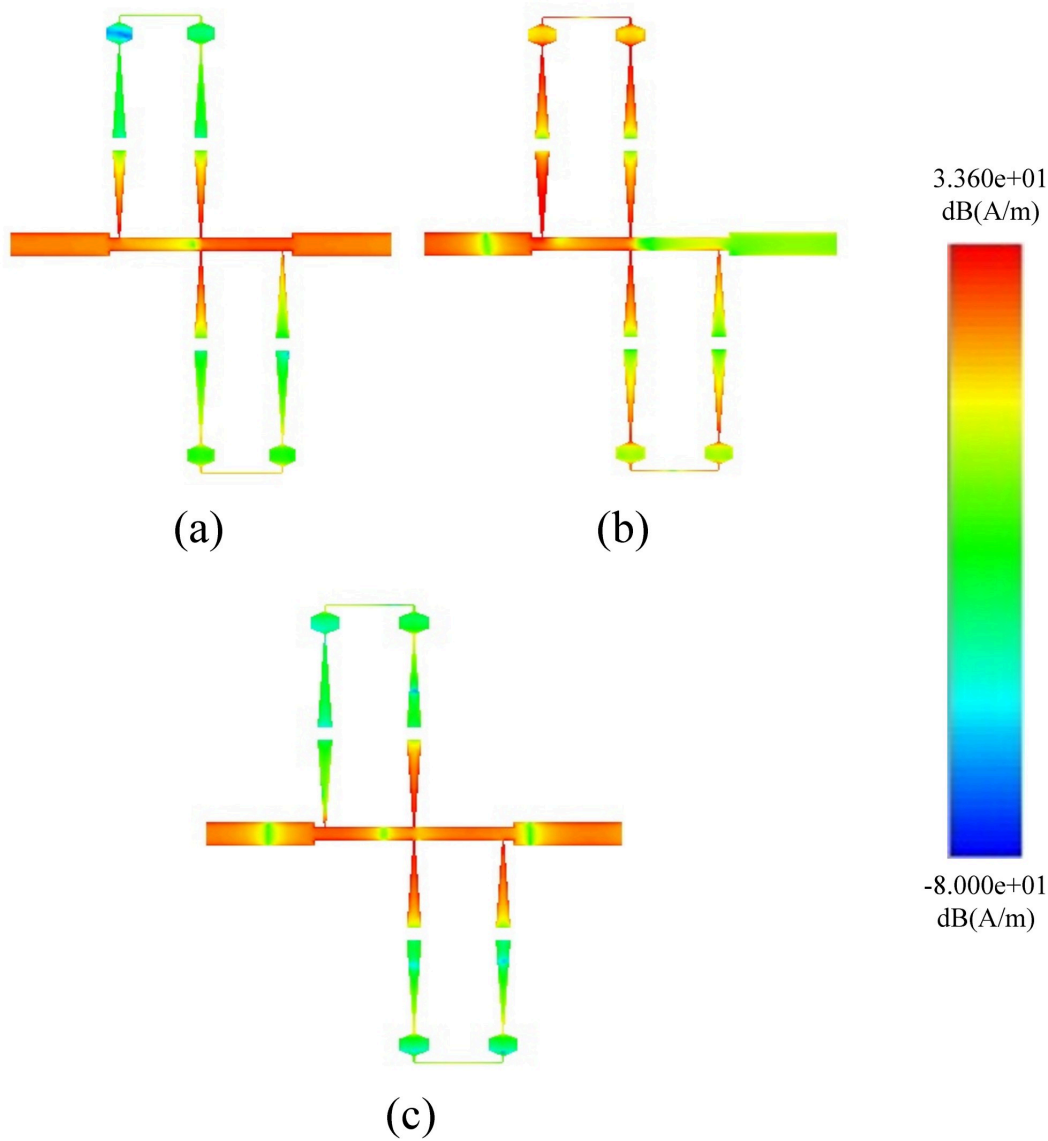
Current distribution of second-order BSF.

**Fig 12:**
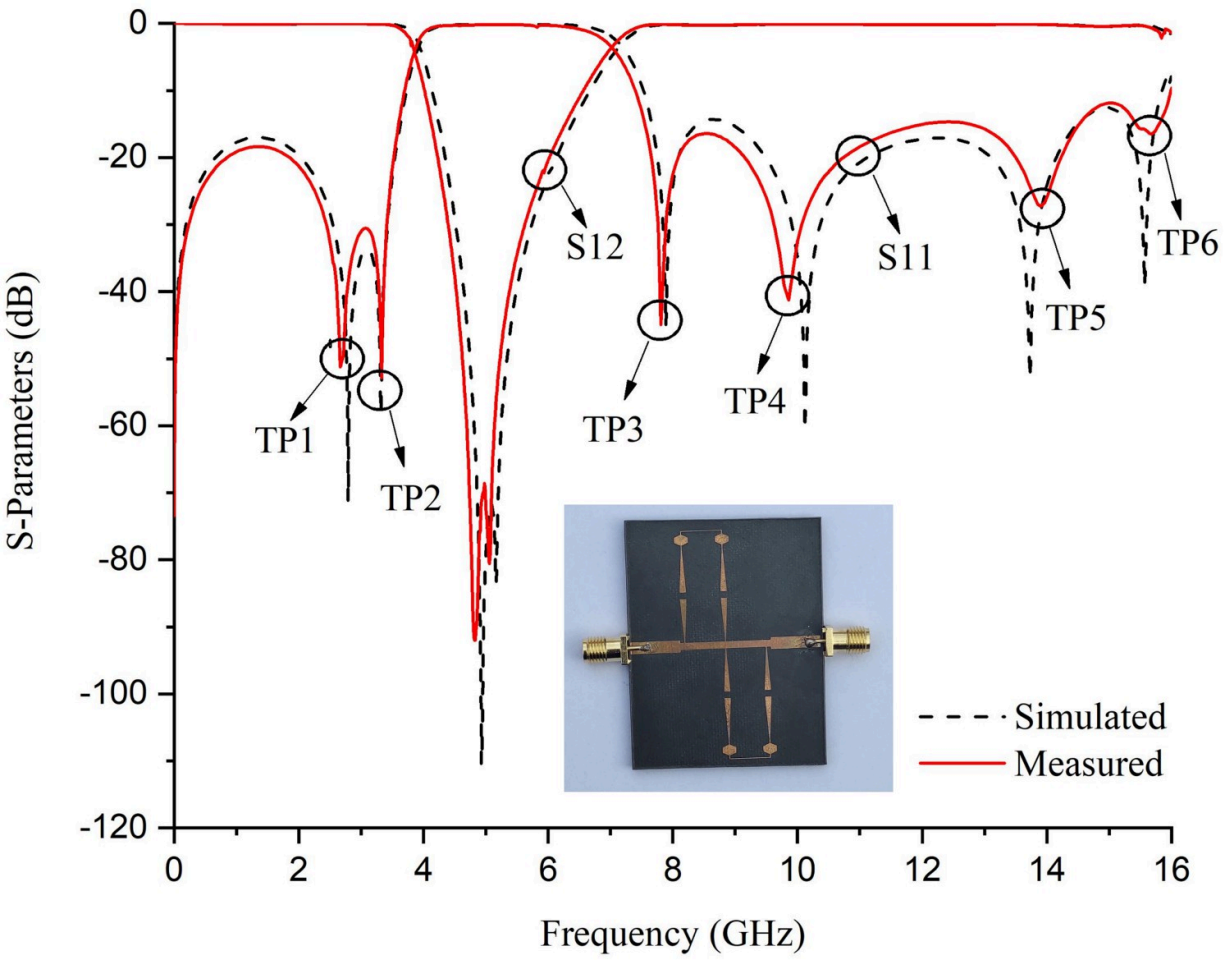
S-Parameter response of the fabricated second-order BSF.

Figs 11 and 12 shows the Electrical Field Spectrum and fabricated prototype with S-Parameter response of second-order dual-path SIR structure having the same physical parameters. By increasing the order of the proposed BSF, two transmission zeros are measured with an increased rejection level. Furthermore, six transmission poles have been measured around the stopband and located at 2.94, 3.3, 7.9, 10.1, 13.6, and 15.56 GHz. It is also worth mentioning that by increasing the order of proposed BSF, the selectivity around the stopband has also been increased.

**Fig 13:**
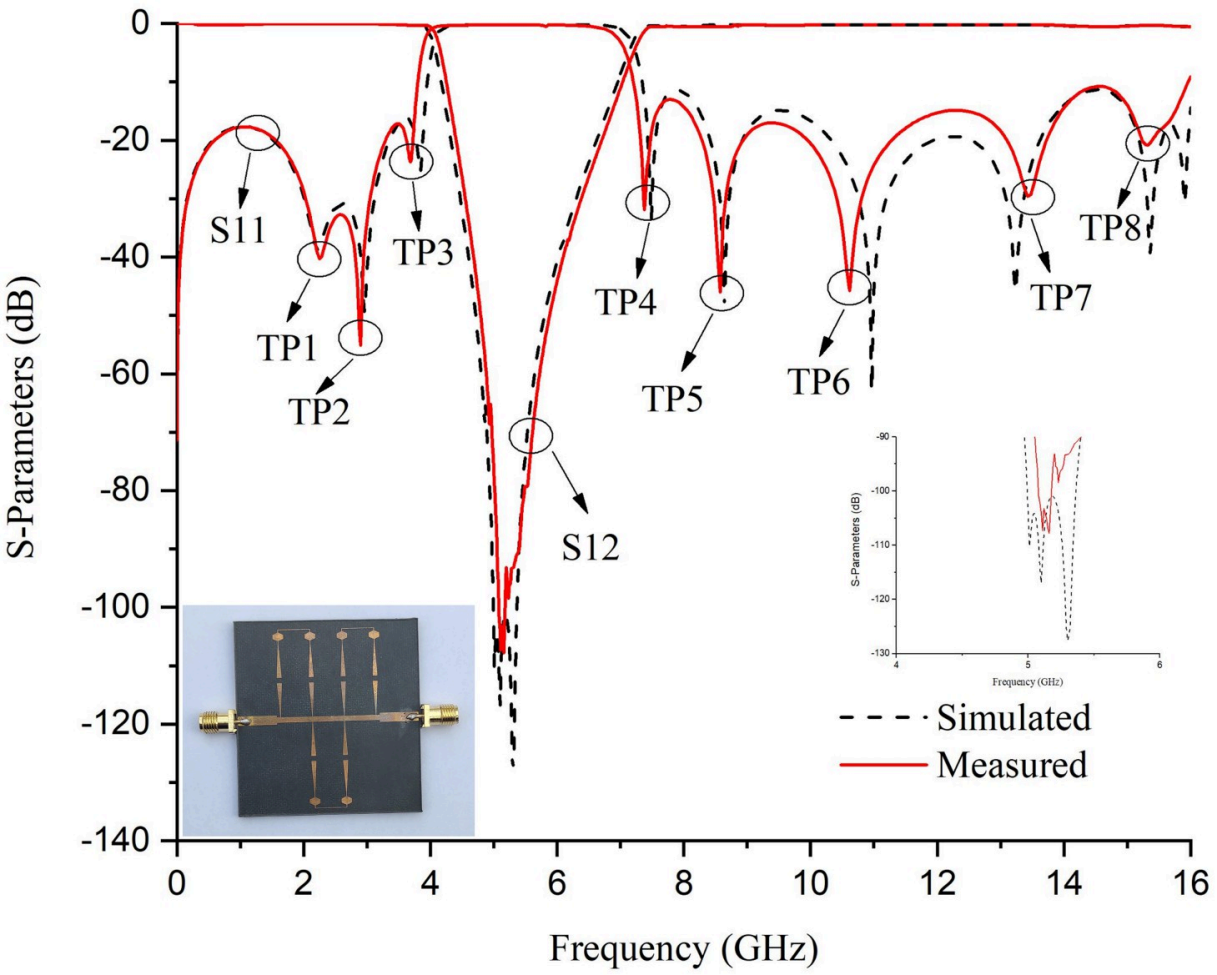
S-Parameter response of the fabricated third-order BSF.

**Fig 14:**
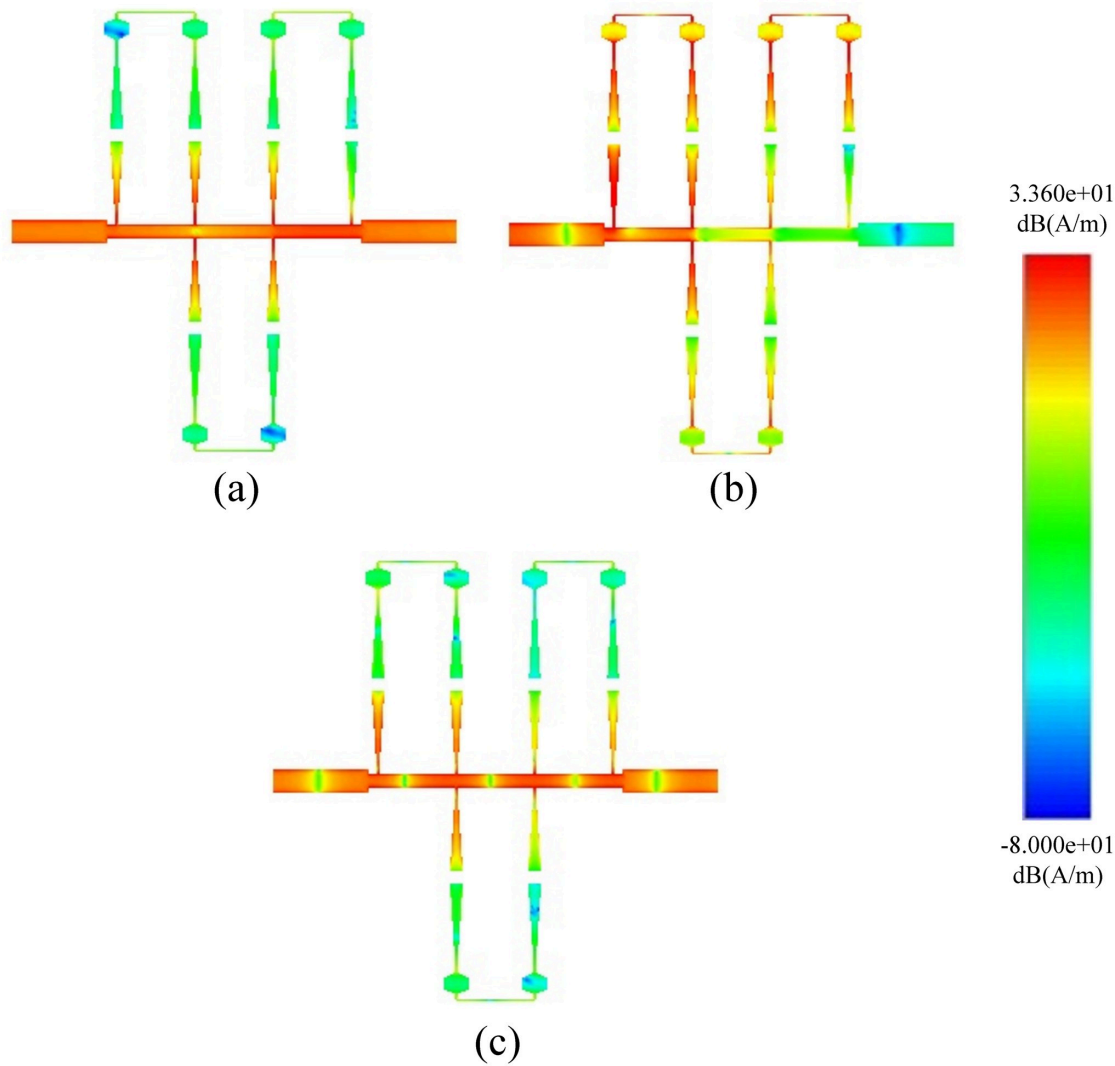
Current distribution of third-order BSF.

In addition, by increasing the order of the proposed BSF to three, as shown in Fig 13, the transmission zeros increase to three, meanwhile the rejection level also increase to 104.3 dB, which further improves the selectivity. Here, it is also notable that the third-order BSF provides nine transmission poles around the stopband, which are located at 2.21, 2.97, 3.7, 7.46, 8.62, 10.93, 13.15, 15.24, and 15.82 GHz respectively. The measured out of stopband return loss is greater than 11 dB. The electrical field spectrum shown in Fig 14 validate the design concept of dual-path SIR structure, where stopband is due to the destructive interference of both paths.

Table 1 illustrates the measured results of all the fabricated prototypes. Comparing all the prototypes, prototype 3 supersede all other two prototypes in term of electrical performance.

**Table 1:** Comparison between proposed three protypes.

Fabricated Topology	Transmission Zeros(TZ’s)	Transmission Poles(TP’s)	Out-of-Band return-loss(*S*_11_)	Maximum Rejection Level	10dB-Fractional bandwidth (FBW)
Prototype 1	1	4	14.8 dB	62.4 dB	29.8%
Prototype 2	2	6	12.7 dB	71.8 dB	51%
Prototype 3	3	9	11.9 dB	104.3 dB	58.5%

Moreover, the out-of-band harmonic suppression is greater than 11.9 dB. By comparing prototype 3 with the other reported topologies shown in Table 2, we have obtained a notably high selective response of the bandstop filter at high frequency.

**Table 2:** Comparison between previous studies and proposed work.

Ref	Center-Frequency	TZ’s	TP’s	Minimum Out of BRL (*S*_11_)	Minimum IL	FBW	Circuit size (*λ*_*g*_×*λ*_*g*_)
[2]	1	5	4	24 dB	17 dB	64%	0.78 ×.28
[5]	1.5	4	3	≥20 dB	≥10 dB	122.5%	0.36 ×.25
[7]	1.5	5	4	10 dB	≥50 dB	122.5%	0.59 ×.10
[8]	1.05	1	4	20 dB	26 dB	55.24%	0.5 ×.35
[9]	1.5	5	3	≥20 dB	≥35 dB	113.4%	0.44 ×.09
[14]	2.45	3	N/A	N/A	≥40 dB	3.2%	0.14 ×.66
[15]	1.9	1	3	≥15 dB	≥30 dB	40%	N/A
This Work	5.25	3	9	11.9 dB	104.3 dB	58.5%	0.77 × 0.7

## 3 Conclusion

This paper presents a novel compact, highly selective wideband bandstop RF filter based upon a dual path (CCSIR) structure. The designed filter operates at 5.25 GHz with fractional bandwidth of up to 58.5%. Three transmission zeros and nine transmission poles ensure good electrical performance. It has been observed that the selectivity can be improved by increasing the order of the dual coupled step impedance resonator. Three prototypes were fabricated and measured to validate the theoretical model. It is concluded that the suggested BSF can play a crucial role in rejecting unwanted signals with high attenuation levels. Furthermore, the proposed wideband BSF is a viable choice for next generation applications due to its flexible design and out-of-band insertion level up to 16 GHz. The fabricated prototype of third order BSF has a compact size of (0.7 × 0.77)λ_*g*_ at 5.25 GHz.
